# Prediction of benzimidazole therapy duration with PET/CT in inoperable patients with alveolar echinococcosis

**DOI:** 10.1038/s41598-022-15641-5

**Published:** 2022-07-06

**Authors:** Lars Husmann, Hannes Gruenig, Caecilia S. Reiner, Ansgar Deibel, Bruno Ledergerber, Virginia Liberini, Stephan Skawran, Urs J. Muehlematter, Michael Messerli, Barbara Hasse, Beat Muellhaupt, Martin W. Huellner

**Affiliations:** 1 Department of Nuclear Medicine, University Hospital Zurich, Raemistrasse 100, 8091 Zurich, Switzerland; 2 Institute of Diagnostic and Interventional Radiology, University Hospital Zurich, University of Zurich, Zurich, Switzerland; 3 Division of Gastroenterology and Hepatology, University Hospital Zurich, University of Zurich, Zurich, Switzerland; 4 Division of Infectious Diseases and Hospital Epidemiology, University Hospital Zurich, University of Zurich, Zurich, Switzerland; 5 Nuclear Medicine Unit, Department of Medical Science, University of Turin, Turin, Italy

**Keywords:** Hepatology, Molecular medicine

## Abstract

Alveolar echinococcosis is a rare parasitic disease, most frequently affecting the liver, as a slow-growing tumor-like lesion. If inoperable, long-term benzimidazole therapy is required, which is associated with high healthcare costs and occasionally with increased morbidity. The aim of our study was to determine the role ^18^F-fluorodeoxyglucose (FDG) positron emission tomography/computed tomography (PET/CT) in staging of patients with alveolar echinococcosis and to identify quantitative imaging parameters related to patient outcome and/or duration of benzimidazole therapy. In this single-center retrospective cohort study, 47 PET/CT performed for staging in patients with confirmed alveolar echinococcosis were analysed. In 43 patients (91%) benzimidazole therapy was initiated and was successfully stopped after a median of 870 days (766–2517) in 14/43 patients (33%). In inoperable patients, tests for trend of survivor functions displayed clear trends for longer benzimidazole therapy duration (p = 0.05; n = 25), and for longer time intervals to reach non-detectable serum concentration of Em-18 antibodies (p = 0.01, n = 15) across tertiles of SUVratio (maximum standardized uptake value in the echinococcus manifestation compared to normal liver tissue). Hence, in inoperable patients with alveolar echinococcosis, PET/CT performed for staging may predict the duration of benzimidazole therapy.

## Introduction

Alveolar echinococcosis is a rare parasitic disease, observed only in the northern hemisphere with a main endemic region in Europe in eastern France, southern Germany, Austria and Switzerland. The incidence of alveolar echinococcosis in Switzerland is 0.26 cases per 100,000 population per year^1^. In the initial phase, alveolar echinococcosis is most frequently diagnosed by liver imaging, where the disease usually presents as a slow-growing tumor-like lesion. The prognosis for untreated alveolar echinococcosis is poor, and radical surgical resection of the parasitic liver lesion is the treatment of choice, since complete cure may thereby be achieved^2^. If the disease is confined to the liver, curative surgery should be performed with a safe distance of at least 1 mm (R0 resection) followed by two years of anthelmintic treatment, which provides a good chance of long term disease-free survival^3^. In patients after incomplete surgical resection (i.e., R1 and R2 resections) or with advanced inoperable stages, lifelong anthelmintic medication is recommended. For these patients, the introduction of benzimidazole therapy has led to a major improvement in survival^4–8^. However, lifelong therapy is associated with high healthcare costs and occasionally with increased morbidity, as side effects are common. In general, benzimidazole therapy is considered parasitostatic^9^. However, numerous reports have shown, that after an undefined length of therapy, treatment effects may be parasitocidal, and treatment may occasionally be safely discontinued in such cases^10,11^. Hence, accurate pretherapeutic staging in alveolar echinococcosis is essential to determine whether curative surgery is feasible. Computed tomography (CT), magnetic resonance imaging (MRI), and ultrasonography are established imaging modalities for staging in alveolar echinococcosis^12^. 18F-fluorodeoxyglucose (FDG) positron emission tomography/computed tomography (PET/CT) offers the additional possibility to assess and quantify the viability of echinococcosis manifestations, and we hypothesize, that quantitative imaging parameters of PET/CT may predict the necessary duration of benzimidazole therapy.

Therefore, the aim of our study was to determine the role of FDG-PET/CT in staging of patients with alveolar echinococcosis in an endemic region in Switzerland and to identify quantitative imaging parameters related to patient outcome and/or duration of benzimidazole therapy.

## Methods

### Study design and data collection

This retrospective study included consecutive patients referred to PET/CT for staging of alveolar echinococcosis between the years 2005 and 2019 at the University Hospital of Zurich. Imaging data of MRI and contrast-enhanced CT was included in the analysis if performed within a time frame of three months before or after the PET/CT.

Clinical follow-up was performed in all patients by reviewing electronic patient charts. We collected all relevant clinical data (such as patient demographics, laboratory data, and clinical and treatment information) at the time of initial diagnosis, at discontinuation of benzimidazole therapy, and at the last recorded clinical visit (last follow-up December 2020).

The local ethics committee, namely the Kantonale Ethikkomission Zürich, approved the study protocol (BASEC-Nr. 2018-01855) and all patients examined between 2016 and 2019 signed written informed consent to the retrospective use of their clinical data for research. For patients scanned between the years 2005 and 2015, informed consent was waived, due to retrospective inclusion by the local ethics committee, namely the Kantonale Ethikkomission Zürich (study protocol BASEC-Nr. 2018-01855). All procedures were performed in accordance with the 1964 Helsinki declaration and its later amendments or comparable ethical standards.

### Imaging data acquisition

All imaging examinations followed basic study protocols. For PET/CT, patients fasted for at least four hours, FDG dosage was body-weight adjusted, the uptake time was standardized to 60 min in supine position, a non-enhanced CT scan was performed and used for attenuation correction, and data was acquired with arms overhead whenever possible. Body weight, height, and blood glucose level were measured prior to imaging, and blood glucose levels < 12 mmol/l were accepted^13^. Five different types of PET/CT scanners were used throughout the study period, i.e. Discovery STE, Discovery LS, Discovery RX, Discovery MI, and Discovery 690 (all GE Healthcare, Waukesha, WI). To compensate for differences in the sensitivity of the different PET/CT scanner generations, we measured the metabolic activity in normal/non-infected liver tissue and in the mediastinal blood pool for reference.

Contrast-enhanced CT was performed of the chest and/or abdomen after intravenous injection of 80 ml iodinated contrast material (Visipaque^®^ 320, GE Healthcare), timed for imaging at the portal venous phase with a tube voltage of 120 kV and a tube current–time product of 100–320 mAs.

MRI examinations were performed following a standard liver MRI protocol, which included at least T2-weighted sequences with and without fat saturation in axial and/or coronal plane and T1-weighted sequences with fat saturation before and after i.v. contrast administration in arterial (typically 30 s), portal venous (typically 60–90 s) and delayed phases (typically 120–240 s). Extracellular contrast agents were used from different vendors. Imaging was either performed at 1.5 or 3.0 T (Aera, Avanto or Skyra, Siemens Healthineers, Siemens, Erlangen, Germany; GE Signa HDxt or GE MR750w, GE Healthcare, Waukesha, WI; Ingenia or Achieva, Philips Healthcare, Best, the Netherlands) using dedicated phased array channel coils.

### Image analysis and definitions

All PET/CT and contrast-enhanced CT data sets were retrospectively reanalysed in consensus by two experienced and doubly board certified nuclear medicine physicians and radiologist on a dedicated workstation (Advantage Workstation, Version 4.6; GE Healthcare Biosciences, Pittsburgh, PA). All MRI data were reanalysed by a radiologist and a doubly board certified nuclear medicine physician and radiologist in consensus. Readers were blinded to all clinical patient outcome data, and collected the following data:

For PET/CT, contrast-enhanced CT, and MRI, readers quantified the number of detectable hepatic and extrahepatic lesions, and the size of the largest lesion. With PET/CT and contrast-enhanced CT, the extent of disease was staged (i.e. PNM stage (P = parasitic mass in the liver; N = involvement of neighboring organs; M = metastasis) as defined by the WHO Informal Working Group on Echinococcosis^2,14–16^, the CT findings were classified according to the EMUC-CT classification^17,18^, and the presence and pattern of lesion calcifications was determined.

MRI findings were classified, according to the recommendations by Kodoma et al.^19^ (i.e. type 1: multiple small cysts without a solid component; type 2: multiple small cysts with a solid component; type 3: a solid component surrounding a large and/or irregular cysts with multiple small cysts; type 4: a solid component without cysts; type 5: a large cyst without a solid component).

For contrast-enhanced CT and MRI, the presence of contrast enhancement was rated, using a four-point score (score 1: no enhancement; score 2: no clear enhancement; score 3; suspicion of enhancement; score 4: clear contrast enhancement).

Furthermore, quantitative imaging parameters were measured in PET/CT, i.e. maximum and peak standardized uptake value (SUVmax and SUVpeak) of FDG, in the largest and/or most FDG-avid manifestation as well as in non-infected liver tissue (the latter for reference). SUVmax is defined as the maximum intensity voxel, SUVpeak as the average activity concentration within a 1 cm^3^ spherical volume of interest centered on the hottest voxel, both within a defined subspace of the PET image matrix.

Finally, serum samples were tested at the Institute of Parasitology, University of Zurich. EMII/3–10 or its derivative EM-18 were used, which are encoded by part of the EM-10 gene sequence, and the EM-VF Western blot for serological confirmation of alveolar echinococcosis^10^.

### Patient follow-up

Clinical follow-up of all patients was performed by reviewing electronic patient charts. Patient data were recorded at the time of staging and at the last recorded clinical visit (latest retrospective follow-up in December 2020). Patient demographics, laboratory data, and clinical information were assessed for all patients, including data on patient survival, duration of benzimidazole therapy and time to reach no detectable levels of Em-18 antibodies.

### Statistical analyses

Statistical analyses were performed using commercially available software (Stata/SE, Version 16.1, StataCorp, College Station, TX). Variables were expressed as median and IQR (25th, 75th percentiles) or percentages. We used tests for trend of survivor functions to assess the association of tertiles of SUVmax, SUVpeak, and SUVratio, the number and maximum size of echinococcus manifestations, the five categories of the KODOMA score, and the four categories of the EMUC CT classification with the duration of benzimidazole therapy and the duration to reach no detectable levels of Em-18. We used the log-rank test to assess the association of curative versus non-curative surgery with the duration of benzimidazole therapy and the duration to reach no detectable levels of Em-18. Kaplan–Meier estimates were used to describe survival from date of diagnosis to last clinical follow-up or death at 1 and 5 years. A p-value of ≤ 0.05 was considered to indicate statistical significance.

## Results

### Patient population

PET/CT for staging was performed in 47 patients with serology-confirmed alveolar echinococcosis. An additional contrast-enhanced CT of the abdomen or chest and abdomen was performed in 35 patients, and an additional MRI of the liver was performed in 26 patients. Demographics of the patient population are displayed in Tables 1, 2 and 3.

**Table 1:** Patient demographics.

Number of patients	47
Median age in years (IQR)	57 (45–66)
Median weight in kg (IQR)	60 (53–133)
Female gender, n (%)	34 (72%)
Number of curative operations, n (%)	13 (28%)

**Table 2:** Patient demographics of all patients with curative operations of alveolar echinococcosis.

ID	Age	PNM			Initial clinical symptoms	Number of lesions	Size of biggest lesion (mm)	SUV ratio	FDG uptake pattern	CE score in CT	CE score in MRI	EMUC-CT	KODOMA	Duration* and status of follow-up (days)	Duration** and status of benzimidazole therapy (days)
01	39	P2	N0	M0	None	2	37	2.8	Inhomogeneous	2	4	IIIA−	1	772/alive	780/completed
02	54	P1	N0	M0	Abdominal pain	1	56	2.3	Multifocal	na	3	I−	2	1627/alive	859/completed
03	73	P2	N0	M0	None	1	21	2.7	Focal	2	na	I−	na	2070/alive	694/completed
04	45	P1	N0	M1	n.a	2	99	2.0	Ringlike	na	na	II−	na	1911/alive	1530/completed
05	29	P2	N0	M0	Jaundice	1	51	1.4	Inhomogeneous	4	na	I−	na	4993/alive	751/completed
06	36	P3	N0	M0	Jaundice	1	52	4.0	Inhomogeneous	3	na	I+	na	4018/alive	1006/completed
07	64	P4	N0	M0	Jaundice	1	121	1.7	Inhomogeneous	2	1	II+	3	3755/alive	881/completed
08	24	P4	N1	M0	Abdominal pain	1	135	1.6	Ringlike	2	1	II+	1	1288/alive	949/completed
09	61	P4	N0	M0	Jaundice	1	71	2.6	Inhomogeneous	2	3	I−	4	1942/alive	1010/completed
10	56	P1	N0	M0	Weight loss	1	55	1.0	Ringlike	na	na	II−	na	578/alive	467/completed
11	58	P1	N1	M0	Back pain	1	65	2.7	Ringlike	1	1	II+	1	2089/alive	785/completed
12	73	P2	N0	M0	Abdominal pain	1	104	2.0	Ringlike	na	1	IIIA+	3	64/alive	24/ongoing
13	32	P4	N0	M0	n.a	2	45	2.0	Focal	4	na	II+	na	1841/alive	761/completed

*ID* patient identification, *PNM* (PNM stage: P = parasitic mass in the liver, N = involvement of neighboring organs, M = metastasis), *SUV* standardized uptake value, *FDG* ^18^F-fluorodeoxyglucose, *CE score* contrast enhancement score (1: no enhancement, 2: most likely no enhancement, 3: suspicion for enhancement, 4: enhancement), *CT* computed tomography, *MRI* magnetic resonance imaging, *EMUC-CT* EMUC-CT classification^17^, *KODOMA* KODOMA-classification^19^, *na* not applicable; *since PET/CT; **since initiation of therapy.

**Table 3:** Patient demographics of all inoperable patients with alveolar echinococcosis.

ID	Age	PNM			Initial clinical symptoms	Number of lesions	Size of biggest lesion (mm)	SUV ratio	FDG uptake pattern	CE score in CT	CE score in MRI	EMUC-CT	KODOMA	Duration* and status of follow-up (days)	Duration** and status of benzimidazole therapy (days)
01	81	P1	N0	M0	None	1	41	1.0	Inhomogeneous	1	1	II−	1	1621/alive	1601/ongoing
02	55	P2	N0	M0	Abdominal pain	2	39	1.5	Ringlike	1	1	II−	2	1110/alive	1131/ongoing
03	57	P4	N1	M0	None	1	62	1.7	Ringlike	2	3	I+	1	921/dead^1^	1052/completed
04	57	P4	N1	M0	Abdominal pain	1	62	2.0	Focal	1	1	II+	1	2488/alive	2524/ongoing
05	48	P1	N0	M0	None	1	37	1.3	Focal	na	na	II−	na	3771/alive	3621/ongoing
06	64	P4	N1	M0	Abdominal pain	15	42	2.0	Focal	na	4	II+, IV	2	1552/alive	1544/ongoing
07	38	P3	N0	M0	Jaundice	1	31	1.6	Inhomogeneous	na	1	II+	5	4340/alive	2517/completed
08	64	P1	N0	M0	None	1	42	1.2	Focal	1	na	II+	na	2177/alive	74/completed
09	46	P4	N1	M1	Edema	2	73	2.8	Ringlike	2	na	II+	na	3880/alive	3898/ongoing
10	59	P3	N1	M0	Diplopia	6	124	1.8	Inhomogeneous	2	na	II+	na	5508/alive	5586/ongoing
11	25	P3	N0	M0	na	> 50	44	1.2	Inhomogeneous	na	na	I−, IV	na	4570/alive	1944/completed
12	66	P3	N0	M0	Fever	3	32	2.5	Ringlike	2	1	II−	3	2198/dead^2^	2206/ongoing
13	87	P3	N0	M0	Abdominal pain	8	121	2.0	Ringlike	2	na	II+	na	1447/alive	241/completed
14	51	P4	N0	M0	Abdominal pain	1	120	2.1	Inhomogeneous	2	na	I−	na	3726/alive	3735/ongoing
15	39	P4	N1	M0	Abdominal pain	4	77	1.5	Focal	2	1	II+	3	3852/alive	3954/ongoing
16	76	P3	N0	M1	None	1	142	2.0	Ringlike	2	na	II+	na	1105/dead^3^	1080/ongoing
17	62	P1	N1	M0	None	3	36	0.8	No uptake	na	na	IIIA+	na	3559/alive	na
18	75	P1	N0	M0	Abdominal pain	20	49	0.6	No uptake	na	na	IIIA, IV	na	1835/alive	na
19	62	P4	N1	M0	None	1	132	1.9	Ringlike	1	na	IIIB−	na	883/dead^4^	749/ongoing
20	60	P1	N0	M0	None	9	43	0.6	No uptake	na	3	II+, IV	2	2092/alive	na
21	67	P3	N1	M0	Abdominal pain	2	95	3.2	Inhomogeneous	2	4	II+	1	2096/alive	2075/ongoing
22	67	P1	N0	M0	None	5	13	0.7	No uptake	1	1	IV	5	2150/alive	na
23	32	P4	N1	M0	Abdominal pain	4	61	3.6	Inhomogeneous	3	na	II−	na	1597/alive	1633/ongoing
24	41	P2	N1	M0	None	1	107	2.5	Ringlike	2	3	II+	3	945/alive	945/ongoing
25	72	P4	N1	M1	None	1	184	4.6	Ringlike	2	4	IIIB−	3	1333/alive	1328/ongoing
26	77	P2	N1	M1	None	2	78	3.7	Inhomogeneous	2	4	I+	2	412/dead^4^	413/ongoing
27	51	P4	N1	M1	Jaundice	2	110	3.0	Inhomogeneous	3	4	I+	3	938/alive	927/ongoing
28	63	P2	N0	M0	None	2	27	1.3	Inhomogeneous	na	1	II+	1	1958/alive	565/completed
29	71	P1	N0	M0	None	1	67	2.6	Ringlike	2	1	II+	3	1386/alive	1325/ongoing
30	28	P1	N0	M1	None	4	25	1.3	Ringlike	1	3	IV	3	1427/alive	721/ongoing
31	57	P4	N1	M1	None	17	84	3.8	Ringlike	4	na	IV	na	1422/alive	1412/ongoing
32	73	P2	N0	M0	None	2	115	2.5	Ringlike	2	1	II+	1	558/alive	565/ongoing
33	54	P2	N0	M0	None	2	56	1.4	Inhomogeneous	1	3	II+	1	554/alive	542/ongoing
34	56	P2	N1	M0	None	1	77	1.1	Ringlike	1	na	II−	na	514/alive	492/ongoing

*ID* patient identification, *PNM* (PNM stage: P = parasitic mass in the liver, N = involvement of neighboring organs, M = metastasis), *SUV* standardized uptake value, *FDG* ^18^F-fluorodeoxyglucose, *CE score* contrast enhancement score (1: no enhancement, 2: most likely no enhancement, 3: suspicion for enhancement, 4: enhancement), *CT* computed tomography, *MRI* magnetic resonance imaging, *EMUC-CT* EMUC-CT classification^17^, *KODOMA* KODOMA-classification^19^, *na* not applicable; *since PET/CT; **since initiation of therapy; ^1^due to thyroid cancer; ^2^due to sepsis; ^3^due to leukemia; ^4^unknown cause of death.

At the time of the PET/CT examination, benzimidazole therapy was already started in 16 (34%) patients [for 23 days (8–398)]. In four (9%) patients, no benzimidazole therapy was initiated throughout the study period (all asymptomatic patients with no detectable levels of Em-18 antibodies and negative PET/CT). Thirteen patients (28%) were operated with a curative approach (Table 2), while 34 patients (72%) were deemed inoperable (Table 3).

### PET/CT

**Figure 1:**
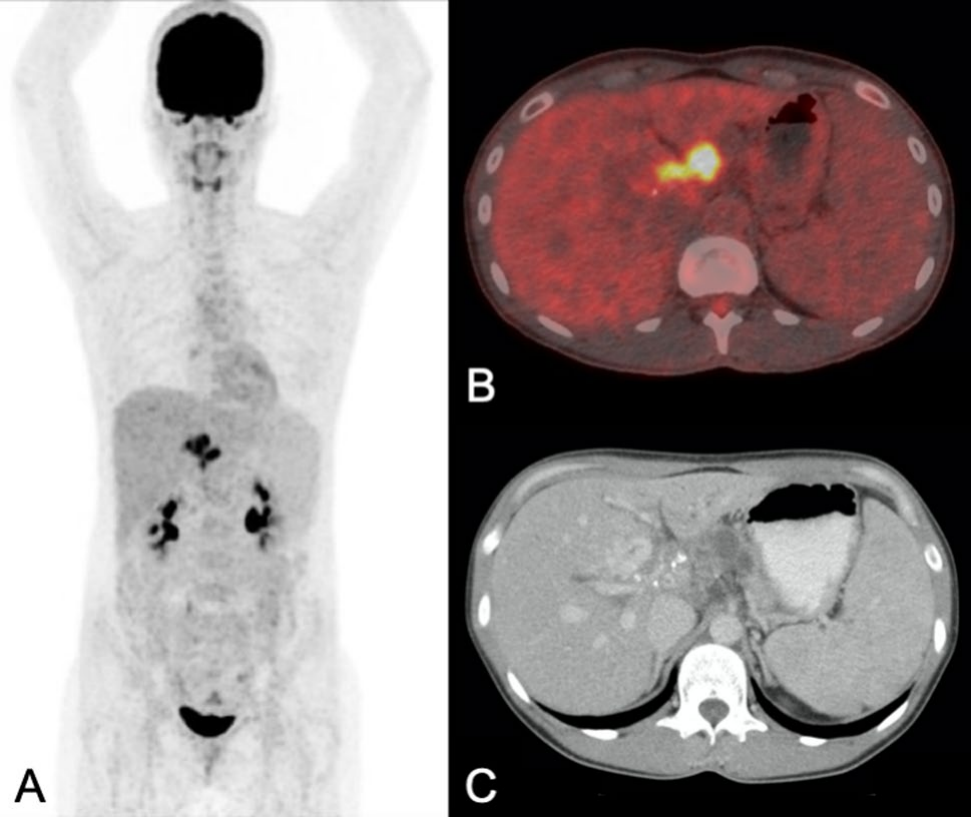
PET/CT performed for staging of alveolar echinococcosis in a 37-year old woman (patient 23 in Table 3) showed intense metabolic activity [in maximum intensity reconstructions of PET (**A**) and fused PET/CT images (**B**)] in a hilar lesion infiltrating the pancreas (dilated pancreatic duct on axial contrast-enhanced CT images in (**C**). SUVmax was 11.1 in the echinococcosis manifestation, and 3.1 in non-infected liver tissue; SUVratio was 3.6. After 1458 days of benzimidazole therapy, levels of Em-18 antibodies became undetectable, but therapy was not yet stopped at the end of the present study period (in total after 1633 days of therapy).

**Figure 2:**
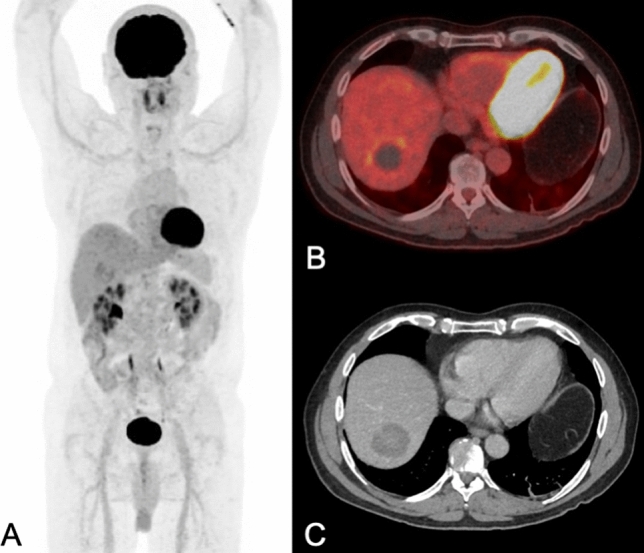
PET/CT performed for staging of alveolar echinococcosis in a 55-year old man (patient 02 in Table 3) showed mild metabolic activity [in maximum intensity reconstructions of PET (**A**) and fused PET/CT images (**B**)] at the margins of a round and hypodense lesions in the right liver lobe [as shown on axial contrast-enhanced CT images in (**C**)]. SUVmax was 4.7 in the echinococcosis manifestations, and 3.2 in non-infected liver tissue; SUVratio was 1.5. After 1000 days of benzimidazole therapy, levels of Em-18 antibodies became undetectable, but therapy was not yet stopped at the end of the present study period (in total after 1131 days of therapy).

PET/CT was successfully performed with diagnostic image quality after intravenous injection of FDG (i.e., 296 Megabecquerel (interquartile range (IQR) 223–392) in all 47 patients with alveolar echinococcosis (Figs. 1 and 2). Median SUVmax in echinococcosis manifestations with the highest FDG-uptake in all patients was 5.4 (3.7–11.8). Median SUVmax of non-infected liver tissue was 3.1 (2.5–9.0). The median SUVratio between echinococcosis manifestations and non-infected liver tissue was 2.0 (1.4–4.6). All individual values are displayed in Tables 2 and 3. Respective values for median SUVpeak were 4.3 (2.8–8.9) in echinococcosis manifestations, 2.2 (1.9–5.7) in non-infected liver tissue, and 1.9 (1.4–3.7) for the ratio of SUVpeak.

### Contrast-enhanced CT and MRI

The disease extent was classified according to the EMUC-CT classification^17,18^ for CT and according to the recommendations by Kodoma et al.^19^ for MRI, and data for each patient is given in Tables 2 and 3, if the respective examination was performed. Furthermore, data on contrast-enhancement is given for both imaging modalities in Tables 2 and 3.

### Outcome

All patients were clinically followed for a median of 5.3 years (IQR 3.3–7.0) after their initial diagnosis and for a median of 5.1 years (IQR 3.1–6.1) after their staging PET/CT examination. The overall mortality was 1.8 per 100 patient years (95% confidence interval [CI] 0.7–4.3). The one-year and five-year freedom from all-cause mortality was 100% (CI 100–100), and 90% (CI 76–96), respectively. Five events occurred in 277.6 years of follow-up time; i.e. three patients (6%) died because of reasons considered unrelated to alveolar echinococcosis (thyroid cancer, leukemia, sepsis), in two patients (4%) the cause of death remained unknown (Table 3).

### Benzimidazole therapy

In 43/47 patients (91%) with alveolar echinococcosis, benzimidazole therapy was initiated. Therapy was prematurely stopped due to adverse effects in 4/43 (9%) patients (i.e. after 74, 241, 565 and 1052 days). Four patients died during ongoing therapy (Table 3). In 14/43 (33%) patients, benzimidazole therapy was successfully stopped after a median of 870 days (766–2517), thereof 12 were operated with a curative approach and two were not operated.

In six patients benzimidazole therapy started > 30 days prior to the staging PET/CT examination (i.e. 36, 37, 80, 89, 404, and 2037 days), 10 patients started < 30 days prior to PET/CT [median 8 days (7–30)], and 27 patients started a median of 22 days (9–706) after PET/CT.

In four of the patients (9%) with negative PET/CT findings at staging, no benzimidazole therapy was initiated, and no detectable levels of antibodies were recorded throughout the entire study period. Overall 18 of 47 patients (38%) had no detectable levels of Em-18 antibodies (or its predecessor EmII/3–10) at the time of initial diagnosis; in 8 patients (17%) the data for the initial level of antibodies was unavailable, and 21 had positive levels of antibodies (45%).

### Probability to stop benzimidazole therapy

**Figure 3:**
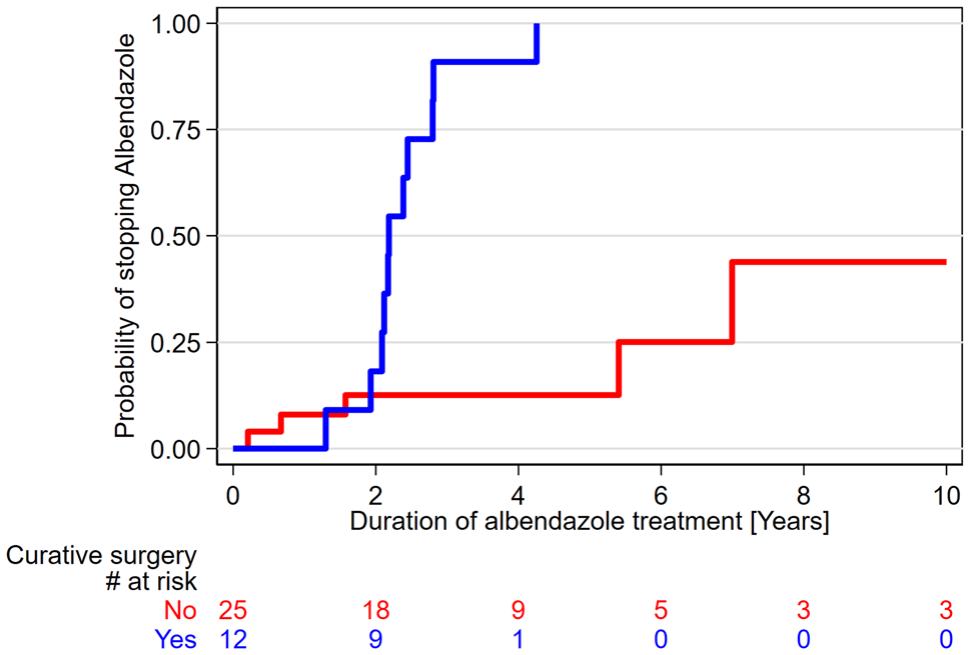
The probability of stopping benzimidazole (i.e. albendazole) therapy is higher if patients underwent surgery with curative intent (Log-rank test p < 0.0001).

To determine the probability to stop benzimidazole therapy, we excluded all patients never treated with benzimidazole within the study period (n = 4), and all patients who received benzimidazole therapy for longer than 30 days prior to the PET/CT examination (n = 6). Hence, 37 patients were included in this analysis, 12 were operated, while 25 were not. The probability to stop benzimidazole therapy was significantly higher (p < 0.001) in operated patients (Fig. 3).

**Figure 4:**
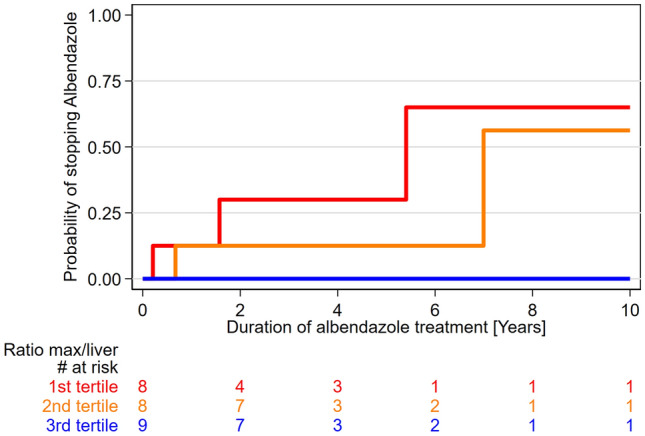
The graph displays a trend (test for trend of survivor function; p = 0.05) toward higher probability of stopping benzimidazole (i.e. albendazole) therapy over time in patients with lower SUVratio (i.e., median SUVratio in the first, second and third tertile were 1.23, 2.01 and 3.03).

To determine whether the number, the maximum size and/or the metabolic activity in the echinococcosis manifestations measured in the staging PET/CT examination were related to the probability of stopping benzimidazole therapy, we additionally excluded all operated patients. Hence, 25 patients were included in this subanalysis, showing a clear trend for longer benzimidazole therapy duration across tertiles of SUVratio (p = 0.05; Fig. 4). Respective p-values for SUVmax, SUVpeak, SUVpeak ratio, and the number and the maximum size of the echinococcosis manifestations were not significant (0.56, 0.24, 0.08, 0.38, and 0.21). Meaningful analyses of the KODOMA and EMUC CT classification were not feasible because of very skewed distributions.

### Probability to reach no detectable levels of Em-18 antibodies

To determine the probability of reaching no detectable levels of Em-18 antibodies, we excluded all patients never treated with benzimidazole within the study period (n = 4), all patients who received benzimidazole therapy for longer than 30 days prior to the PET/CT examination (n = 6), and all patients with no available data (n = 8) or no detectable levels of Em-18 antibodies at the time of initial diagnosis (n = 10). Hence, 19 patients were included in this analysis, 4 were operated, while 15 were not. The probability of reaching no detectable levels of Em-18 was significantly higher (p < 0.001) in operated patients.

To determine whether the number, the maximum size and/or the metabolic activity in the echinococcosis manifestations measured in the staging PET/CT examination were related to the probability of reaching no detectable levels of Em-18, we additionally excluded all operated patients. Hence, 15 patients were included in this subanalysis, showing a significant trend for longer time intervals for reaching no detectable levels of Em-18 across tertiles of SUVratio (p = 0.011); the median time to reach no detectable levels of Em-18 was 3.8 years (95% CI 2.4–n.a.). The respective p-values for trend for the other parameters were: 0.047 for SUVpeak ratio, 0.089 for SUVmax, 0.30 for SUVpeak, 0.84 for the number, and n.s. for the maximum size of the echinococcosis manifestations. Meaningful analyses of the KODOMA and EMUC CT classification were not feasible because of very skewed distributions.

## Discussion

We analysed the role of PET/CT in staging of patients with alveolar echinococcosis. Our study results are: (i) time to reach no detectable levels of Em-18 antibodies and the duration of benzimidazole therapy is significantly shorter in patients operated with a curative intent. (ii) in staging of inoperable patients, SUVratio (a quantitative imaging parameter derived from PET/CT) is associated with the time to reach no detectable levels of Em-18 antibodies and with the duration of benzimidazole therapy. (iii) clinical outcome was excellent, i.e. the 1-year and 5-year freedom from all-cause mortality was 100%, and 90%, respectively (five events occurred in 278 years of follow-up time; i.e. in three patients the cause of death was considered unrelated to alveolar echinococcosis, in two patients it remained unknown).

With the introduction of benzimidazole therapy, survival rates of patients with alveolar echinococcosis have become comparable to normal patient populations^20^. In our study population, five events occurred in 278 years of follow-up, and none could be clearly attributed to alveolar echinococcosis as the cause of death. Similarly, a previous publication^21^ with a more selected patient population (i.e. patients were only included, if PET/CT was negative and levels of Em-18 antibodies were not detectable at the end of benzimidazole treatment) described two events, occurring in 292 patient years of follow-up, and these events also could not be clearly related to alveolar echinococcosis.

As an alternative measure of patient outcome, we analyzed the time to reach no detectable levels of Em-18 antibodies and the duration of benzimidazole therapy and its relation to curative surgery and quantitative PET/CT parameters, acquired at initial staging of the disease.

Curative surgery of metabolically active alveolar echinococcosis is generally combined with two years of benzimidazole treatment^22^. Accordingly, in all operated patients of the present study who were followed for at least two years, benzimidazole treatment was successfully stopped and Em-18 antibodies dropped to undetectable levels.

In metabolically inactive alveolar echinococcosis a watch and wait strategy may be suggested^22^. In line with this strategy, four patients of the present study cohort were not treated with benzimidazole and did not develop detectable levels of Em-18 antibodies throughout the whole study period.

In inoperable, metabolically active alveolar echinococcosis long-term benzimidazole treatment is recommended, and may be discontinued two years after echinococcus manifestations become metabolically inactive on PET/CT and Em-18 antibodies become not detectable^21,22^. To the best of our knowledge, the relation of the time interval to reach undetectable levels of EM-18 and the duration of benzimidazole therapy with the metabolic activity in PET/CT (i.e. SUVratio of the most FDG-avid liver lesion) has not been evaluated previously. Our results underline the hypothesis, that in inoperable patients, the metabolic activity in manifestations of alveolar echinococcosis in PET/CT performed for staging, is associated with the time to reach no detectable levels of Em-18 antibodies and with the duration of benzimidazole therapy. Hence, PET/CT may be used to predict the duration of benzimidazole therapy in patients with alveolar echinococcosis in the future.

### Limitations of the study

A rather small number of patients was examined with all three imaging modalities (i.e. PET/CT, contrast-enhanced CT and MRI) in the present retrospective study of a rare disease. Furthermore, the study population was heterogeneous, including inoperable patients, patients undergoing curative surgery and patients who were not treated at all. However, after defining several exclusion criteria to preclude bias, we were able to perform statistically reliable subanalyses on patient outcome in inoperable patients with alveolar echinococcosis, in which PET/CT was performed for staging.

## Conclusion

In inoperable patients with alveolar echinococcosis, PET/CT performed for staging may predict outcome, as the quantitative imaging parameter SUVratio is associated with the time to reach no detectable levels of Em-18 antibodies and with the duration of benzimidazole therapy. Furthermore, in selective patients with undetectable levels of Em-18 antibodies, negative PET/CT findings at staging may allow for a watch and wait strategy.
